# Genome‐wide association study in diverse Iranian wheat germplasms detected several putative genomic regions associated with stem rust resistance

**DOI:** 10.1002/fsn3.2082

**Published:** 2021-01-10

**Authors:** Ali Saremirad, Mohammad Reza Bihamta, Ali Malihipour, Khodadad Mostafavi, Hadi Alipour

**Affiliations:** ^1^ Plant breeding Ph. D. student Department of Agronomy and Plant Breeding Young Researchers and Elite Club Karaj Branch Islamic Azad University Karaj Iran; ^2^ Department of Agronomy and Plant Breeding Faculty of Agriculture University of Tehran Karaj Iran; ^3^ Cereal Research Department, Seed and Plant Improvement Institute (SPII) AREEO Karaj Alborz Iran; ^4^ Associate Professor Department of Agronomy and Plant Breeding Karaj Branch Islamic Azad University Karaj Iran; ^5^ Department of Plant Breeding and Biotechnology Faculty of Agriculture Urmia University Urmia Iran

**Keywords:** association mapping, *Puccinia graminis* f. sp. *tritici*, *Sr* genes, wheat

## Abstract

Stem rust is one of the most important diseases, threatening global wheat production. Identifying genomic regions associated with resistance to stem rust at the seedling stage may contribute wheat breeders to introduce durably resistant varieties. Genome‐wide association study (GWAS) approach was applied to detect stem rust (*Sr*) resistance genes/QTLs in a set of 282 Iranian bread wheat varieties and landraces. Germplasms evaluated for infection type and latent period in four races of *Puccinia graminis* f. sp. *tritici* (*Pgt*). A total of 3 QTLs for infection type (R^2^ values from 9.54% to 10.76%) and 4 QTLs for the latent period (R^2^ values from 8.97% to 12.24%) of studied *Pgt* races were identified in the original dataset. However, using the imputed SNPs dataset, the number of QTLs for infection type increased to 10 QTLs (R^2^ values from 8.12% to 11.19%), and for the latent period increased to 44 QTLs (R^2^ values from 9.47% to 26.70%). According to the results, the Iranian wheat germplasms are a valuable source of resistance to stem rust which can be used in wheat breeding programs. Furthermore, new information for the selection of resistant genotypes against the disease through improving marker‐assisted selection efficiency has been suggested.

## Graphical abstract

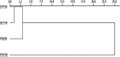

## 1 INTRODUCTION

Wheat (*Triticum* *aestivum* L.) is a major staple food crop in the world. It is predicted that the cereal yield should increase by 50% to meet the food demand of the human population by 2050 (Godfray et al., 2010). Wheat production and yield are constantly challenged by various biotic and abiotic stresses. Diseases and pests are among the most important biotic stresses resulting in approximately 20% losses of the global wheat yield annually (Oerke, 2006). Rusts are among the most damaging cereal diseases which have coexisted and evolved during the domestication of cereals (Schafer et al., 1984). Black stem rust of wheat caused by *Puccinia graminis* Pers. f. sp. *tritici* (*Pgt*) is considered a very serious threat in major wheat‐growing regions around the world. This disease, which rapidly develops under humid conditions and high temperatures (15–35ºC), is the most damaging rust among wheat rusts and the extent of damage may result in the large‐scale destruction across wheat fields (Roelfs et al., 1992; Singh et al., 2015).

Fungicide application and stem rust‐resistant wheat cultivars are two important strategies to combat *Pgt*. However, the fungicide application may not always be feasible due to the high cost, risk of fungicide resistance, and environmental contamination. Using resistant wheat cultivars is the most effective and economical way to control the disease. In this regard, having sufficient knowledge on population genetics of the disease causal agent and identifying effective resistance genes in the selected wheat genotypes are of great importance.

Plant disease resistance is generally divided into monogenic and polygenic categories based on the mode of inheritance in plants. In the monogenic type, a gene is responsible for the expression of resistance, and the type of resistance, dominance, and codominance, as well as discrete variation, can be obtained in differentiating generations and can be identified using the Mendelian laws and the monosomic technique of the genes. In the polygenic type, resistance is governed by several genes, so monosomic techniques and Mendelian laws cannot be used to identify resistance genes. Linkage mapping to detect quantitative traits loci (QTLs) is used as a methodology to understand the genetic control of polygenic traits. The major limitations of this methodology are a small number of crossovers, resulting in low genetic mapping resolution (10–20 cM) and the high cost of population replication to reach sufficient crossovers. Besides, the created populations are only useful for limited traits and studies (Gupta et al., 2005; Holland, 2007). An alternative methodology with the advantage of using natural populations is association mapping.

Association mapping seeks to identify continuous markers linked to one or more quantitative traits. It is based on the assumption that the observed phenotypic variation is related to genetic variation. In this method, a large and diverse set of individuals/lines drawn from a population is randomly collected and mapping is done based on linkage disequilibrium. Association mapping has been used for both whole‐genome scanning and candidate gene analysis (Kraakman et al., 2004; Pasam et al., 2012; Rafalski & Morgante, 2004; Rostoks et al., 2006; Thornsberry et al., 2001). Association mapping is much more accurate than linkage mapping due to its many recombinations (Moose & Mumm, 2008). It is also more compatible with genetically diverse germplasm and allows the mapping of several traits simultaneously. Therefore, there is no need to create two‐parental populations for each trait, which in turn incurs additional costs for genotypic and phenotypic evaluation.

The objectives of this study were to carry out a genome‐wide search in Iranian wheat genotypes for resistance loci to *Pgt* races at the seedling stage, and the identification of genomic regions suitable for marker‐assisted selection and further genetic dissection.

## 2 MATERIALS AND METHODS

### 2.1 Plant materials

A set of 282 Iranian bread wheat genotypes, including 193 landraces collected between 1931 and 1968 and 89 commercial varieties released between 1942 and 2014 were selected to form a stem rust AM panel. Landraces were obtained from the Gene Bank of the University of Tehran and commercial varieties from Seed and Plant Improvement Institute (SPII), Karaj, Alborz, Iran, and Dryland Agricultural Research Institute (DARI), Maragheh, East Azarbaijan, Iran. A detailed description of the AM panel is attached as the additional file 1, Table S1, and S2.

### 2.2 Phenotypic evaluation

Phenotypic evaluation of AM panel against *Pgt* was carried out at the Cereal Research Department, Seed and Plant Improvement Institute (SPII), Karaj, Alborz, Iran, in 2019. The AM panel was evaluated under greenhouse conditions using a randomized complete block design with two replications for each of the four *Pgt* races. Seeds of each genotype were planted in pots with 10 cm height and diameter containing 1:1:2 ratio of common soil, peat moss, and leaf mold. After 8–10 days, when the first leaf of the seedlings well developed (12 stage of phenological development based on the Zadoks et al. (1974) scale), the spores of four stem rust races were collected from different regions of Iran (Table 1) were inoculated separately. The inoculated plants were kept in a dark chamber for 24 hr at 18 ± 2°C and near saturation moisture and then transferred to a 22 ± 2°C greenhouse with a 16‐hr photoperiod. Inoculated plants were examined from the second day to record the latent period (LP). Fourteen days after inoculation, seedling infection type (IT) was recorded using the 0 to 4 scale introduced by Stakman et al. (1962) and modified by McIntosh et al. (1995) where ITs of 0, 1, and 2 were considered as low ITs, and ITs of 3, and 4 were considered as high ITs.

**TABLE 1:** Detailed description of the Pgt races used to evaluate the wheat genotypes

Isolate	Location	Race	Stem rust resistance (*Sr*) genes	
			Ineffective	Effective
94–8	Boroujerd, Lorestan, Iran	TTTTF	*Sr5, Sr6, Sr7a, Sr7b, Sr8a, Sr8b, Sr9a, Sr9b, Sr9d, Sr9e, Sr9g, Sr10, Sr11, Sr12, Sr13, Sr14, Sr15, Sr16, Sr17, Sr18, Sr19, Sr20, Sr21, Sr22, Sr23, Sr25, Sr28, Sr29, Sr30, Sr34, Sr35, Sr36, Sr37, Sr38, Sr40, SrTmp,* and *SrMcN*	*Sr24, Sr26, Sr27, Sr31, Sr33,* and *Sr39*
94–15	Kelardasht, Mazandaran, Iran	PTRTF	*Sr5, Sr6, Sr7b, Sr8a, Sr8b, Sr9a, Sr9b, Sr9d, Sr9e, Sr9g, Sr10, Sr11, Sr12, Sr13, Sr14, Sr15, Sr16, Sr17, Sr18, Sr19, Sr20, Sr25, Sr27, Sr28, Sr29, Sr34, Sr35, Sr36, Sr37, Sr38, Sr39, SrTmp,* and *SrMcN*	*Sr7a, Sr21, Sr22, Sr23, Sr24, Sr26, Sr30, Sr31, Sr32, Sr33,* and *Sr40*
95–2	Shavour, Khouzestan, Iran	TTKTK	*Sr5, Sr6, Sr7a, Sr7b, Sr8a, Sr8b, Sr9a, Sr9b, Sr9d, Sr9e, Sr9g, Sr10, Sr11, Sr12, Sr14, Sr15, Sr16, Sr17, Sr18, Sr19, Sr20, Sr21, Sr23, Sr28, Sr29, Sr30, Sr31, Sr33, Sr34, Sr37, Sr38, SrTmp,* and *SrMcN*	*Sr13, Sr22, Sr24, Sr25, Sr26, Sr27, Sr32, Sr35, Sr36, Sr39,* and *Sr40*
95–31	Kelardasht, Mazandaran, Iran	TKTTF	*Sr5, Sr6, Sr7a, Sr7b, Sr8a, Sr8b, Sr9a, Sr9b, Sr9d, Sr9e, Sr9g, Sr10, Sr11, Sr12, Sr14, Sr15, Sr16, Sr17, Sr18, Sr19, Sr20, Sr21, Sr23, Sr28, Sr29, Sr30, Sr34, Sr35, Sr36, Sr37, Sr38, SrTmp,* and *SrMcN*	*Sr22, Sr24, Sr25, Sr26, Sr27, Sr31, Sr32, Sr33, Sr39,* and *Sr40*

### 2.3 Phenotypic data analysis

Before performing the analysis of variance, the hypotheses of variance analysis were examined and the results confirmed the hypotheses. To calculate genetic parameters, analysis of variance was performed for the IT and LP of the studied *Pgt* races. Genetic, environmental, and phenotypic variances were calculated using the Comstock & Robinson (1952) method, using Equations 1, 2, and 3, respectively:

(1) $$\sigma_{g}^{2}=\frac{MS_{g}‐MS_{e}}{r}$$

(2) $$\sigma_{e}^{2}=MS_{e}$$

(3) $$\sigma_{p}^{2}=\sigma_{g}^{2}+MS_{e}$$

In these equations, MS_g_ is genotype mean square, MS_e_ is mean square error and r is the number of experimental replications. Heritability was estimated by the Falconer (1989) method through the 4 equation:

(4) $$h^{2}=\frac{\sigma_{g}^{2}}{\sigma_{p}^{2}}$$

where $\sigma_{g}^{2}$ is the genetic variance and $\sigma_{p}^{2}$ is the phenotypic variance obtained from the variance analysis table according to Comstock & Robinson (1952) method. To estimate the coefficient of genetic and phenotypic variation, Singh and Chaudhary (1985) method was used based on 5 and 6 equations:

(5) $$\text{GCV}\;\left(\text{\%}\;\right)=\frac{\sqrt{\sigma_{g}^{2}}}{\bar{x}}\times 100$$

(6) $$\text{PCV}\;\left(\text{\%}\;\right)=\frac{\sqrt{\sigma_{p}^{2}}}{\bar{x}}\times 100$$

where $\bar{x}$ is the average of trait. Genetic efficiency was also calculated according to the Allard (1960) and Singh and Chaudhary (1985) methods using the (7) equation:

(7) $$GA=k\times \sigma_{p}\times h^{2}$$

where *k* is 10% of the selection pressure (1.75), $\sigma_{p}$ is the phenotypic standard deviation and *h* ^2^ is the heritability.

A combined analysis of variance was performed using SAS software 9.3, and genetic parameters were calculated using Excel. The race grouping was performed based on Ward's method using SPSS software.

### 2.4 Genotyping by sequencing and imputation method

The development and sequencing of a GBS library for the Iranian wheat have previously been described by Alipour et al. (2017). The genomic library was constructed according to the method of Poland et al. (2012). For this purpose, first, the extracted DNA was normalized and then enzymatic digestion was performed with two enzymes *PstI* and *MspI*. Then, barcode adapters were attached to each sample, so that each sample has a different barcode. To remove extras except for barcoded genomic DNA, purification was performed by QIAquick (Qiagen) PCR purification kits. Finally, the amplified fragments were selected on an E‐gel system with a size between 300–250 bp and sent for sequencing by the Ion Proton System. Sequencing data were treated for 64 bp, and the same reads were grouped into tags. Identical sequence tags were aligned to identify the SNPs within the tags and the SNPs were summoned using the UNEAK (Universal Network Enabled Analysis Kit) GBS pipeline (Lu et al., 2013) as part of the TASSEL 4.0 bioinformatics package (Bradbury et al., 2007). SNPs with heterozygosity > 10%, minor allele frequency (MAF) <5%, and missing data > 20% were eliminated to reduce false‐positive error results. After specifying the haplotype phase for all individuals, the data were subjected to imputation using BEAGLE v3.3.2 (Browning & Browning, 2009) based on available allele frequencies obtained. During imputation, four different reference genomes were assessed, among which the W7984 reference genome was shown to have the greatest imputation accuracy (Alipour et al., 2019). The different chromosomes LD decay was obtained using the ggplot2 package in RStudio (Team, 2015) based on LOESS regression.

### 2.5 Population structure and kinship matrix

The population structure of the AM panel was investigated using 8,959 SNP markers for the original dataset, and 43,921 SNP markers for the imputed dataset, which were distributed across the 21 wheat chromosomes, using the software STRUCTURE v2.3.3 (Pritchard et al., 2000). The optimal number of *K* was determined by an admixture model and with a burn‐in and simulation phase consisting of 10,000 steps for values of *K = *1 to 10. The *ΔK* statistic was used to determine the most desirable subpopulation number, and its plot was plotted for consecutive *K* values. The observed and expected values allele frequencies were used to estimate the linkage disequilibrium between markers in TASSEL v.5 (Bradbury et al., 2007). Then, population structure matrix *Q* (*n* × *p*), where *n* is the number of assayed genotypes and *p* is the number of defined subpopulations, was used in association studies. Cluster analysis and principal component analysis were also performed to determine the genetic relationships between the AM panel.

### 2.6 Genome‐wide association study

A genome‐wide association study for loci governing *Pgt* resistance was conducted using phenotypic data converted to a linear scale. To use the modified Stakman et al. (1962) ITs in the genome‐wide association studies (GWAS), the 0 to 4 scale was converted to a 1 to 13 linear. Both the general linear model (GLM) and mixed linear model (MLM) were used to examine the accurate association between marker and trait in TASSEL v.5 (Bradbury et al., 2007). The GAPIT package (Lipka et al., 2012) was also used to perform association mapping in both GLM and MLM methods in RStudio (Team, 2015). The results of TASSEL and GAPIT were investigated using a *t* test. According to the results, it was found that the general linear model derived from TASSEL provides more accurate information about the marker–trait association.

### 2.7 Gene annotation

Sequences surrounding of significant SNP markers were obtained from the wheat 90 K SNP database (Wang et al., 2014) and used for assessing gene annotation using Gramene (http://www.gramene.org/) by aligning them to the IWGSC RefSeq v1.0 annotation (https://wheat‐urgi.versailles. inra.fr/Seq‐Repository/Annotations). The function of putative genes was explored by investigating the pathways in which the encoded enzymes were involved in. After aligning SNPs sequences to the reference genome, overlapping genes with the highest identity percentage and blast score were selected for further processing. The ontology of each adjacent genes with *Triticum aestivum*, including molecular function and biological process, and also orthologous genes in related species were extracted from the ensemble‐gramene database (http://ensembl.gramene.org).

## 3 RESULTS

### 3.1 Phenotypic data analysis

The result of the combined analysis of variance for IT and the LP of wheat genotypes to four *Pgt* races is presented in Table 2. According to the results, the effects of race, genotype, and genotype × race interaction were highly significant for all race's IT and LP.

**TABLE 2:** Combined analysis of variance for infection types and the latent period of wheat genotypes to four *Pgt* races

Source of variation	*df*	Mean squares		Explained variance (%)	
		Infection type	Latent period	Infection type	Latent period
Race	3	145.69**	169.59**	3.97	8.80
Block (race)	4	18.78	3.67	0.68	0.25
Genotype	281	21.20**	8.93**	54.12	43.39
Genotype × race interaction	843	4.74**	2.53**	36.33	36.91
Error	1,124	0.48	0.55	4.90	10.69
Coefficient of variation (%)		6.10	9.58		

** Significant at the 1% probability level.

Genetic parameters including variance components, genetic coefficients, and phenotypic variations, heritability, and genetic efficiency for IT and LP of wheat genotypes against four *Pgt* races were calculated (Table 3). The results showed high genetic and phenotypic variances for IT and LP of wheat genotypes to all races except TKTTF IT and LP. The highest genetic and phenotypic coefficients of variation were obtained for TTKTK IT and LP; the lowest values of these coefficients were detected for TKTTF IT and LP. The heritability and genetic efficiency of the ITs varied from 84.02% to 97.80% and 25.33 to 38.04%, respectively, and for the LPs ranged from 67.68% to 95.34% and 24.85 to 33.55%, respectively.

**TABLE 3:** Amounts of genetic parameters for infection types and latent period collected from the response of wheat genotypes to four *Pgt* races

Genetic parameters	TTTTF		PTRTF		TTKTK		TKTTF	
	IT	LP	IT	LP	IT	LP	IT	LP
Genetic variance	4.131	2.050	4.872	2.050	4.862	2.870	3.380	1.525
Genetic coefficient of variation (%)	17.33	19.087	19.47	19.632	20.66	19.826	15.66	16.034
Phenotypic variance	4.375	2.250	4.981	2.150	5.786	4.240	3.958	1.945
Phenotypic coefficient of variation (%)	17.84	19.987	19.68	20.105	22.54	24.097	16.95	18.107
Environmental variance	0.244	0.200	0.109	0.100	0.924	1.370	0.578	0.420
Heritability (%)	94.43	91.11	97.80	95.34	84.02	67.68	85.40	78.40
Genetic efficiency (%)	29.48	31.87	33.69	33.55	33.14	28.54	25.33	24.85

Abbreviation

IT: Infection type, LP: Latent period.

The frequency of genotypes was calculated based on the 0 to 4 IT scales (McIntosh et al., 1995; Stakman et al., 1962) for each race, and the results were presented in Table 4. Genotypes with seedling response between 0 and 2 (0–0;‐ ;‐ ;1– 1– 1+‐ 2^‐^‐2C‐ 2 and 2^+^) as resistance reaction and genotypes with IT between 3 and 4 (3– 3^+^ and 4) were considered as susceptible reaction (Letta et al., 2014). The results showed that in TTTTF, 16 varieties (17.98%) and 33 landraces (17.10%), in PTRTF, 18 varieties (20.22%) and 40 landraces (20.73%), in TTKTK, 34 varieties (38.20%) and 64 landraces (33.16%), and in TKTTF, 16 varieties (17.98%) and 35 landraces (18.13%) had resistance reaction.

**TABLE 4:** Classification of wheat genotypes based on the reaction to four *Pgt* races

Infection type	Cultivar/ landrace	TTTTF		PTRTF		TTKTK		TKTTF	
		Number	%	Number	%	Number	%	Number	%
Resistance reactions									
0, 0; and ;	Cultivar	–	–	‐	‐	6	6.74	1	1.12
	landrace	2	1.04	3	1.55	5	2.59	1	0.52
;1, 1 and 1^+^	Cultivar	4	4.49	5	6.74	11	12.36	6	6.74
	landrace	12	6.22	13	6.73	17	8.81	10	5.18
2^‐^, 2C, 2 and 2^+^	Cultivar	12	13.48	12	13.48	17	19.10	9	10.11
	landrace	19	9.84	25	12.95	41	21.24	24	12.44
Total	Cultivar	16	17.98	18	20.22	34	38.20	16	17.98
	landrace	33	17.10	40	20.73	64	33.16	35	18.13
Susceptible reactions									
3 and 3^+^	Cultivar	48	53.93	48	53.93	46	51.68	30	33.71
	landrace	63	32.64	82	42.49	106	54.92	58	30.05
4	Cultivar	25	28.09	23	25.84	9	10.11	43	48.41
	landrace	97	50.26	71	36.79	23	11.92	100	51.81
Total	Cultivar	73	82.02	71	79.77	55	61.80	73	82.02
	landrace	160	82.90	153	79.27	129	66.84	158	81.87

**FIGURE 1:**
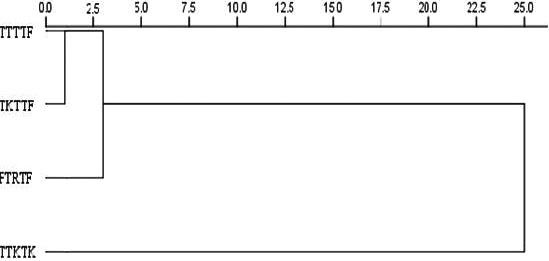
Dendrogram of cluster analysis of four *Pgt* races based on infection types and latent periods of 297 wheat genotypes using the Ward's method

The pattern of diversity among the four *Pgt* races based on Ward's method was shown in Figure 1. The dendrogram obtained from the analysis classified the races into three well‐distinct groups. The TTTTF and TKTTF clustered together while PTRTF and TTKTK showed independent virulence patterns.

### 3.2 Population structure and kinship analysis

**FIGURE 2:**
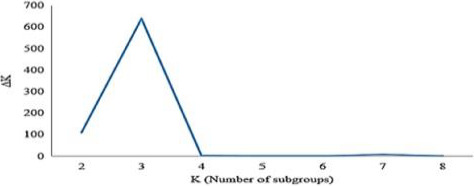
Determination of subpopulation number in wheat genotypes based on ΔK values

**FIGURE 3:**
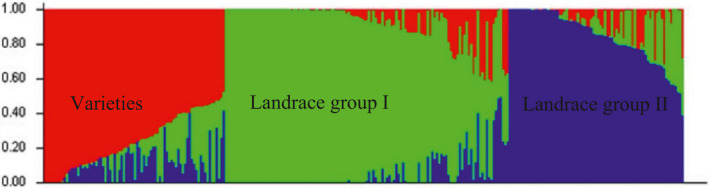
A structure plot of the 282 wheat genotypes and landraces determined by K = 3

To prevent false‐positive results, the AM panel was studied in terms of structure and kinship, and K and ∆K were extracted and their 2D graph was drawn and shown in Figure 2. The graph of ∆K at K = 3 represents the highest value in perfectly specified fracture, dividing the present population into three subpopulations. The allocation of each individual to either group was carried out based on the 70% membership threshold. Details of the subpopulation structure for each of the wheat genotypes are shown in Figure 3. Out of a total of 282 genotypes, 51 genotypes (18.09%) were in the first subpopulation, 103 genotypes (36.52%) were in the second subpopulation and 61 genotypes (21.63%) were in the third subpopulation. The first subpopulation contained 49 landraces and two varieties, the second subpopulation contained 97 landraces and six varieties and the third subpopulation contained 59 varieties and two landraces. When used the imputed SNPs, the studied germplasm has also divided into three subpopulations such as the original dataset that the first subpopulation consisted of 51 genotypes containing 49 landraces and two varieties, the second subpopulation contained 103 genotypes consisting of 97 landraces and six varieties and the third subpopulation contained 61 genotypes consisting of 59 varieties and two landraces.

**FIGURE 4:**
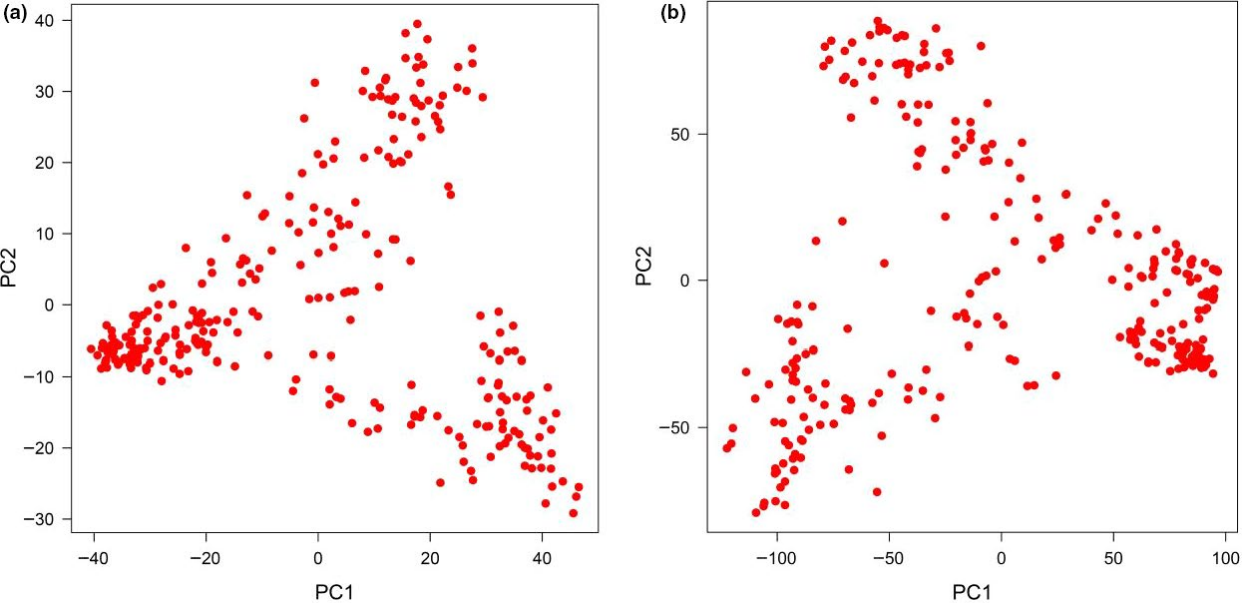
Principal component analysis of the wheat genotypes using original (A) and imputed SNPs (B)

The principal component analysis was performed on the matrix derived from both original and imputed SNPs to further evaluation of population structure and investigation of genetic relationships among wheat genotypes. According to genotypic data, the 2D plot of the original (Figure 4a) and imputed SNPs (Figure 4b) showed almost distinct grouping into three groups. Genotyping variance was explained by the first two main principal components that were 12.70% and 5.95%, respectively, for the original SNPs, and 17.20% and 6.10%, for the imputed SNPs.

**FIGURE 5:**
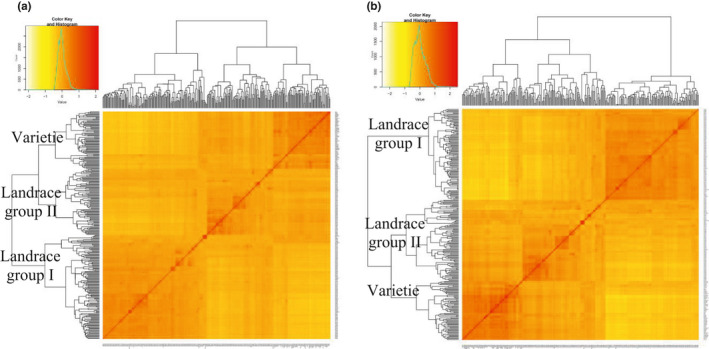
Cluster analysis using Kinship matrix of original (A) and imputed dataset (B) for the wheat genotypes

Group I contains 129 genotypes with 113 landraces and 16 varieties; Group II contains 82 genotypes with 77 landraces and five varieties, and Group III contains 68 varieties and three landraces (Figure 5a). Genotypes also clustered into three main groups when used the imputed SNPs, where Group I contains 111 genotypes with 105 landraces and six varieties; Group II contains 99 genotypes with 85 landraces and 14 varieties; and Group III contains 72 genotypes with 68 varieties and three landraces (Figure 5b). According to the original SNP dataset, 21 varieties appear to be admixed with the two landrace groups, while for the imputed SNP dataset, only 20 such admixed varieties were identified. The admixed varieties originated from Iranian landraces and varieties.

### 3.3 Linkage disequilibrium analysis

Linkage disequilibrium (LD) was examined for wheat varieties and landraces by 404,825 and 40,824 pairwise SNPs, respectively, in the original dataset, and by 2,152,925 SNPs for both wheat varieties and landraces in the imputed dataset. The SNPs were distributed across 21 chromosomes. The range of LD was estimated with a square of allele frequencies (r^2^) between the pairwise markers. LD analysis using the original dataset showed that the average genetic distance for the wheat varieties and landraces was 221.7440 and 221.7461 cM, respectively, and the mean of squared allele‐frequency correlations for them was 0.130 and 0.096, respectively (Table 5). Using an imputed dataset, the average genetic distance was equal for both wheat varieties and landraces (52.82 cM), but their mean of squared allele‐frequency correlation was 0.1924 and 0.1623, respectively (Table 6). The markers were not evenly distributed over different genomes. In the original dataset, the number of markers located on A and B genomes in varieties was equal to the number of markers located on A and B genomes in landraces, but in the D genome, landraces had one less marker than varieties. In the imputed dataset, there was no difference between the number of markers placed on each of the three genomes (A, B, and D) in the varieties with those in the landraces. The B genome had the highest number of markers, whereas the D genome showed the lowest number of markers in the original and imputed dataset. The proportion of each A, B, and D genomes from total pairwise original SNP markers in varieties and landraces were estimated at almost 37, 46, and 17%, respectively, and in the imputed SNP markers approximately 36, 50, and 13%, respectively. Out of 404,825 pairwise SNP markers, 86,415 pairwise markers (21.35%) in varieties and out of 404,824 pairwise SNP markers, 107,976 pairwise SNP markers (26.67%) in landraces showed significant linkage at 1% probability level in the original dataset. According to the imputed dataset, out of 2,152,925 pairwise SNP markers in varieties, 842,785 pairwise SNP markers (39.15%) and out of 2,152,925 pairwise SNP markers in landraces, and 968,270 pairwise SNP markers (44.98%) had significant linkage at 1% probability level.

**TABLE 5:** A summary of observed linkage disequilibrium among pairwise markers and the number of significant pairwise markers per chromosome and genome using the original dataset

Chromosome	Cultivars				Landraces			
	NSP	Distance (cM)	R^2^	SSP	NSP	Distance (cM)	R^2^	SSP
1A	20,325	6.5208	0.1095	4,019	20,325	6.5208	0.0731	4,762
1B	26,450	4.5786	0.1373	5,867	26,450	4.5786	0.0955	8,673
1D	13,350	11.9226	0.1803	3,453	13,350	11.9226	0.1183	4,137
2A	23,950	4.8296	0.1315	5,427	23,950	4.8296	0.1276	8,259
2B	33,350	4.2026	0.1288	8,539	33,350	4.2026	0.0966	9,575
2D	17,050	6.5433	0.2647	4,553	17,050	6.5433	0.1999	5,078
3A	21,500	9.5170	0.0970	3,613	21,500	9.5170	0.0706	4,114
3B	33,050	4.5077	0.1281	7,928	33,050	4.5077	0.0932	9,166
3D	7,550	22.2324	0.0933	701	7,550	22.2324	0.0927	1547
4A	17,450	8.0520	0.1312	3,752	17,450	8.0520	0.1106	4,245
4B	10,600	9.9421	0.0995	1847	10,600	9.9421	0.0527	1,386
4D	3,700	25.9186	0.1359	588	3,700	25.9186	0.1116	1,010
5A	18,250	8.0378	0.1085	3,289	18,250	8.0378	0.0816	4,711
5B	28,850	6.5358	0.1314	7,721	28,850	6.5358	0.0726	6,691
5D	8,350	27.4992	0.0936	805	8,350	27.4992	0.0696	1,394
6A	17,850	7.3937	0.1057	3,279	17,850	7.3937	0.1210	6,715
6B	27,850	4.3847	0.1409	6,565	27,850	4.3847	0.0753	6,593
6D	9,250	18.1532	0.1055	1,397	9,250	18.1532	0.0802	2059
7A	28,550	6.1499	0.1520	5,580	28,550	6.1499	0.1123	8,528
7B	26,800	4.7642	0.1118	5,429	26,800	4.7642	0.0837	6,846
7D	10,750	20.0585	0.1618	2063	10,749	20.0606	0.0932	2,487
A genome	147,875	50.5007	0.1194	28,959	147,875	50.5007	0.0995	41,334
B genome	186,950	38.9156	0.1254	43,896	186,950	38.9156	0.0814	48,930
D genome	70,000	132.3277	0.1479	13,560	69,999	132.3298	0.1094	17,712
Total	404,825	221.7440	0.1309	86,415	404,824	221.7461	0.0968	107,976

Note

NSP: Number of SNP pairwise, SSP: Significant pairwise SNP (*p* < .01).

**TABLE 6:** A summary of observed linkage disequilibrium among pairwise markers and the number of significant pairwise markers per chromosome and genome using the imputed dataset

Chromosome	Cultivars				Landraces			
	NSP	Distance (cM)	R^2^	SSP	NSP	Distance (cM)	R^2^	SSP
1A	110,675	1.3446	0.1392	33,200	110,675	1.3446	0.1079	41,497
1B	149,000	0.9442	0.1923	59,280	149,000	0.9442	0.1497	74,142
1D	47,950	3.5143	0.3028	20,528	47,950	3.5143	0.2257	24,767
2A	136,250	0.8628	0.2672	64,307	136,250	0.8628	0.2766	76,493
2B	185,800	0.7705	0.1841	77,464	185,800	0.7705	0.1554	86,029
2D	67,250	1.6415	0.2201	21,080	67,250	1.6415	0.1777	26,828
3A	95,500	2.3004	0.1487	32,745	95,500	2.3004	0.1212	33,346
3B	199,900	0.7787	0.2307	92,545	199,900	0.7787	0.2029	103,406
3D	35,850	4.6331	0.1521	7,560	35,850	4.6331	0.1547	12,763
4A	129,850	1.3855	0.3447	66,704	129,850	1.3855	0.3293	69,407
4B	59,800	2.2142	0.1378	18,268	59,800	2.2142	0.0968	16,220
4D	13,200	9.3335	0.1573	2,876	13,200	9.3335	0.1239	4,212
5A	71,350	2.0200	0.1620	24,756	71,350	2.0200	0.1344	28,504
5B	151,500	1.3002	0.1888	66,026	151,500	1.3002	0.1377	68,944
5D	31,450	6.9574	0.1470	7,085	31,450	6.9574	0.1286	9,923
6A	98,550	1.3027	0.1721	37,415	98,550	1.3027	0.1688	48,695
6B	188,750	0.6656	0.1834	75,156	188,750	0.6656	0.1275	82,520
6D	37,800	4.1908	0.1357	9,535	37,800	4.1908	0.1358	14,802
7A	147,900	1.1793	0.2230	58,549	147,900	1.1793	0.1951	71,306
7B	148,700	0.9965	0.1464	53,428	148,700	0.9965	0.1154	57,384
7D	45,900	4.4855	0.2044	14,278	45,900	4.4855	0.1436	17,082
A genome	790,075	10.39523	0.2081	317,676	790,075	10.39523	0.1905	369,248
B genome	1,083,450	7.669932	0.1805	442,167	1,083,450	7.669932	0.1408	488,645
D genome	279,400	34.75617	0.1885	82,942	279,400	34.75617	0.1557	110,377
Total	2,152,925	52.82133	0.1924	842,785	2,152,925	52.82133	0.1623	968,270

Note

NSP: Number of Pairwise SNP, SSP: Significant Pairwise SNP (*p* < .01).

### 3.4 Marker–trait association

Table 7 summarized the results of the marker–trait association for the two traits of IT and the LP for each studied *Pgt* race. Totally, 69 and 62 marker–trait associations were identified for IT and the LP of the used races, respectively, in the original dataset with a probability level of 0.1% (*p* ≤ .001). In the imputed dataset, the number of marker–trait associations increased to 504 and 454 for IT and the LP, respectively (*p* ≤ .001). The IT corresponding to all races in the original and imputed dataset had the highest number of the marker–trait association on the B genomes of chromosomes. The TTKTK IT had the same number of the marker–trait association on all three genomes in the original dataset. In the TTTTF, the lowest marker–trait association in original and imputed SNPs in A genome and the other races in the D genome were observed. Regarding the TTTTF, PTRTF, and TKTTF LPs, it was found that the highest number of marker–trait association of original SNPs was located on the B genome and for TTKTK on the A genome. These results were a little different in the imputed SNPs, as the highest number of the marker–trait association was observed for PTRTF and TTKTK in A genome and B genome, respectively. Both the TTTTF and TKTTF had the highest number of marker–trait association located on the B genome in imputed SNPs similar to the results of the original SNPs. Among the ITs of races, TTTTF had 29 and 208 marker–trait associations in the original and imputed SNPs, respectively, and among the LPs of races, the TTKTK race had 19 and 160 marker–trait associations in the original and imputed SNPs, respectively, in the 0.1% probability, which were the highest among the other races.

**TABLE 7:** A summary of marker–trait associations for infection type and the latent period of studied races in Iranian wheat genotypes

Genome	TTTTF		PTRTF		TTKTK		TKTTF	
	IT	LP	IT	LP	IT	LP	IT	LP
	Original data							
Marker–trait association	29	15	15	18	10	19	15	10
Genome A	2	2	2	5	3	6	4	3
Genome B	17	8	9	6	3	2	8	5
Genome D	8	2	1	3	3	8	2	1
Unassembled Chromosomes	2	3	2	4	1	3	1	1
	Imputed data							
Marker–trait association	208	128	109	106	83	160	104	60
Genome A	55	37	27	44	25	53	37	10
Genome B	95	69	67	35	39	75	54	33
Genome D	56	19	13	23	18	29	12	16
Unassembled Chromosomes	2	3	2	4	1	3	1	1

Abbreviation

IT: Infection type, LP: Latent period

The results of the Bonferroni correction at the 5% probability level of the association analysis of original SNPs are presented in Table 8. For Bonferroni correction of the original dataset, 0.05 was divided by the number of the employed markers (8,959 markers) and finally, it was identified that markers were significant at the probability level of 5.58098E‐06. Two QTLs for the TTTTF LP, one QTL for the IT and four QTLs for the LP of PTRTF, one QTL for the IT, and one QTL for the LP of TTKTK, and one QTL for the IT of TKTTF were identified according to the results of Bonferroni correction. The identified QTLs associated with the LP of the studied races, except for one QTL related to the LP of PTTTF located on chromosome 7A with a distance of 133.92 cM, the rest were associated with markers whose genomic location was unknown. The QTL identified for the PTRTF IT was located on chromosome 7A at 132.92 cM. QTL of TTKTK IT was on chromosome 6B at 43.284 cM and QTL of TKTTF IT was on chromosome 1D at 64.821 cM.

**TABLE 8:** Most significant original SNP markers associated with quantitative trait loci for resistance to stem rust races of TTTTF, PTRTF, TTKTK, and TKTTF

Marker	Chr	Pos	P‐value							
			TTTTF		PTRTF		TTKTK		TKTTF	
			IT	LP	IT	LP	IT	LP	IT	LP
rs46629	1D	64.821	—	—	—	—	—	—	1.91E‐6	—
rs23510	6B	43.284	—	—	—	—	3.56E‐6	—	—	—
rs22132	7A	133.92	—	—	3.23E‐6	2.81E‐6	—	—	—	—
rs13588	Un	0	—	1.56E‐6	—	3.38E‐8	—	—	—	—
rs18901	Un	0	—	1.56E‐6	—	3.38E‐8	—	—	—	—
rs27487	Un	0	—	—	—	5.18E‐6	—	2.75E‐7	—	—

Abbreviation

Chr: Chromosome, Pos: Position, IT: Infection type, LP: Latent period, Un: Unassembled Chromosome

Bonferroni correction of the imputed SNPs was performed as with the original SNPs, but with this difference in the number of markers used in this dataset (43,921 markers), 0.05 was divided into these markers number (43,921) and the level of significance was considered to be 1.1384E‐06 and the results are shown in Table 9. In this regard, the TTTTF IT was controlled by six QTLs, which one, two, and one QTLs were located on chromosome 1, chromosome 3, and chromosome 5 of the B genome, respectively; and the other two QTLs were located on chromosome 2D. For the LP of this race (TTTTF), there were 26 QTLs that eight QTLs were on the A genome, 12 QTLs were on the B genome and six QTLs were on the D genome. Chromosomes three, six, and two of the A genomes, chromosomes one, six, three, and two of the B genome, and chromosomes four, seven, and one of the D genome carried QTLs identified in the TTTTF LP. For the IT and LP of PTRTF, 1 and 31 QTLs were, respectively, observed. The only QTL of the IT was present at 4.558 cM of chromosome 3A. From the 31 identified QTLs for the LP, 3, 3, 1, 1, 1, 4, 3, 3, 6, 1, and 3 QTLs were located on chromosomes of 1A, 1B, 1D, 2A, 2B, 3A, 3B, 4D, 6A, 6B, and 7D, respectively; two QTLs were also associated with markers with unknown genomic loci. For the LP of TTKTK, 14 QTLs were detectable, but no QTL was recorded for the IT. The identified QTLs of the LP was located on the A (chromosomes six, three, five, and seven, respectively) and B genomes (chromosomes one and three, respectively), and a QTL was also associated with markers with unknown genomic loci. In the TKTTF, only three QTLs associated with the IT were identified, two QTLs were located on chromosome 1B at 11.376 cM and one QTL was located on chromosome 1D at 64.821 cM.

**TABLE 9:** Most significant imputed SNP markers associated with quantitative trait loci for resistance to stem rust races of TTTTF, PTRTF, TTKTK, and TKTTF

Marker	Chr	Pos	P‐value							
			TTTTF		PTRTF		TTKTK		TKTTF	
			IT	LP	IT	LP	IT	LP	IT	LP
rs63550	1A	44.512	—	—	—	1.15E‐7	—	—	—	—
rs63551	1A	44.512	—	—	—	1.15E‐7	—	—	—	—
rs63552	1A	44.512	—	—	—	1.15E‐7	—	—	—	—
rs12408	1B	54.669	—	1.98E‐7	—	—	—	—	—	—
rs21674	1B	58.08	—	3.47E‐18	—	7.11E‐19	—	1.35E‐10	—	‐
rs45874	1B	11.376	—	—	—	—	—	7.55E‐7	2.11E‐7	—
rs45875	1B	11.376	—	—	—	—	—	7.55E‐7	2.11E‐7	—
rs47978	1B	54.669	—	1.36E‐7	—	2.69E‐7	—	—	—	—
rs51316	1B	25.027	—	8.32E‐9	—	1.12E‐10	—	—	—	—
rs5923	1B	106.992	1.06E‐6	—	—	—	—	—	—	—
rs33527	1D	47.767	—	3.89E‐8	—	8.69E‐8	—	—	—	—
rs46629	1D	64.821	—	—	—	—	—	—	5.21E‐7	—
rs17477	2A	92.517	—	4.31E‐8	—	1.31E‐8	—	—	—	—
rs25281	2B	40.98	—	8.93E‐7	—	2.62E‐8	—	—	—	—
rs46339	2D	79.911	1.00E‐6	—	—	—	—	—	—	—
rs46340	2D	79.911	1.00E‐6	—	—	—	—	—	—	—
rs13329	3A	43.437	—	—	—	—	—	6.96E‐8	—	—
rs50704	3A	4.558	—	5.03E‐8	—	7.21E‐8	—	—	—	—
rs50705	3A	4.558	—	5.03E‐8	—	7.21E‐8	—	—	—	—
rs50706	3A	4.558	—	5.03E‐8	—	7.21E‐8	—	—	—	—
rs62417	3A	4.558	—	—	—	—	—	1.68E‐7	—	—
rs7318	3A	4.558	—	2.55E‐10	6.70E‐8	2.92E‐13	—	—	—	—
rs20274	3B	31.882	5.93E‐7	3.72E‐18	—	1.77E‐19	—	1.11E‐10	—	—
rs20275	3B	31.882	5.93E‐7	3.72E‐18	—	1.77E‐19	—	1.11E‐10	—	—
rs20294	3B	31.882	—	2.75E‐8	—	3.82E‐9	—	—	—	—
rs33960	3B	62.576	—	—	—	—	—	1.78E‐7	—	—
rs65252	4D	54.756	—	2.61E‐8	—	7.93E‐9	—	—	—	—
rs65253	4D	54.756	—	2.61E‐8	—	7.93E‐9	—	—	—	—
rs65254	4D	54.756	—	2.61E‐8	—	7.93E‐9	—	—	—	—
rs51204	5A	93.664	—	—	—	—	—	1.53E‐7	—	—
rs22086	5B	86.610	4.63E‐7	—	—	—	—	—	—	—
rs17868	6A	90.292	—	—	—	2.62E‐7	—	—	—	—
rs34668	6A	50.208	—	2.56E‐18	—	4.91E‐19	—	1.91E‐10	—	—
rs35063	6A	50.208	—	2.21E‐18	—	4.78E‐19	—	1.91E‐10	—	—
rs35074	6A	50.208	—	2.74E‐18	—	4.73E‐19	—	1.95E‐10	—	—
rs4500	6A	10.247	—	—	—	6.97E‐7	—	—	—	—
rs53936	6A	99.391	—	—	—	1.80E‐7	—	—	—	—
rs14828	6B	58.062	—	1.00E‐6	—	—	—	—	—	—
rs14829	6B	58.062	—	1.00E‐6	—	—	—	—	—	—
rs14830	6B	58.062	—	1.00E‐6	—	—	—	—	—	—
rs25306	6B	47.831	—	—	—	6.48E‐8	—	—	—	—
rs32944	6B	47.831	—	4.11E‐8	—	—	—	—	—	—
rs48710	7A	71.904	—	—	—	—	—	8.54E‐8	—	—
rs16240	7D	83.31	—	7.72E‐9	—	4.73E‐9	—	—	—	—
rs20930	7D	83.31	—	1.21E‐8	—	3.81E‐9	—	—	—	—
rs40699	7D	82.173	—	—	—	4.09E‐7	—	—	—	—
rs13588	Un	0	—	—	—	3.38E‐8	—	—	—	—
rs18901	Un	0	—	—	—	3.38E‐8	—	—	—	—
rs27487	Un	0	—	—	—	—	—	2.75E‐7	—	—

Abbreviation

Chr: Chromosome, Pos: Position, IT: Infection type, LP: Latent period, Un: Unassembled Chromosome

Highly significant markers associated with IT and the LP of *Pgt* races, their chromosomal sequence and position, the closest wheat gene or genes to them, orthologous genes (with the highest percentage of identity), identity percentage of the wheat gene or genes that match to the ortholog, molecular function and biological processes of the wheat gene or genes associated with the markers and the extent of phenotype variance explain (R^2^) are reported in Table 10. Out of the 51 highly significant, identified SNP markers, only 9 markers had the adjacent wheat gene or genes with the same chromosomal position. The Ensembl (https://asia.ensembl.org/index.html) site was used to gain further information about adjacent genes beyond the genetic position of the markers. The genes with the highest identity percentage and the lowest E‐value with significant markers were reported. The amount of phenotypic variance explained by SNP markers ranged between 9% and 25%. The rs21674 and rs51316 markers on the 1B justified the high variance explained for the associated traits to other markers. These markers are in the closest genetic position to the TraesCS1B02G208400 and TraesCS1B02G037100 genes, respectively, and each of these genes has a genetic identity with the orthologous genes of AET1Gv20503500 (96.74%) in *Aegilops tauschii* and TRIDC1AG003150 (95) in Triticum dicoccoides. The wheat TraesCS1D02G315800 gene, with an rs46629 marker located on the chromosome 1D, is incomplete identity (100%) with the AET1Gv20751400 ortholog gene of *Aegilops tauschii*.

**TABLE 10:** Basic local alignment search tool results of significant markers for seedling resistance to Pgt races

R2	0.09	0.10	0.25 0.15 0.25	0.09 0.11	0.10	0.09	0.09	0.09	0.12 0.15
Trait	PTRTF (LP)	TTTTF (LP)	TTTTF (LP) PTRTF (LP) TTKTK (LP)	TTTTF (LP) PTRTF (LP)	TTKTK (LP)	TTTTF (IT)	TTTTF (IT)	TKTTF (IT)	TTTTF (LP) PTRTF (LP)
Biological process	—	protein phosphorylation, phosphorylation	regulation of transcription, DNA‐templated	—	protein phosphorylation	—	–	protein processing, oxidation‐reduction process	defense response to bacterium
Molecular function	protein binding	nucleotide‐binding, protein kinase activity, protein serine/threonine kinase activity, protein binding, ATP binding, kinase activity, transferase activity	DNA binding, lipid binding, sequence‐specific DNA binding	protein binding	protein kinase activity, MAP kinase activity, ATP binding	ADP binding	ADP binding	serine‐type endopeptidase activity, superoxide dismutase activity	copper ion binding
Identity	99.70	85.81	96.74	96.94	96.53	74.78	74.78	100	95.00
Orthologous gene	TRIDC6AG012980 ^a^	TRIDC1BG053570 ^a^	AET1Gv20503500 ^b^	AET2Gv20533600 ^b^	TRIDC3BG039070 ^a^	TRIDC6AG061710 ^a^	TRIDC6AG061710 ^a^	AET1Gv20751400 ^b^	TRIDC1AG003150 ^a^
Position (bp)	67,642,014‐67,642,038	555,948,792‐555,952,831	377,964,396‐377,970,306	367,657,356‐367,709,290	418,887,290‐418,898,340	571,193,736‐571,202,194	571,193,736‐571,202,194	410,793,556‐410,798,235	17,730,527‐17,739,883
Adjacent T. aestivum gene	TraesCS6A02G100400	TraesCS1B02G330100	TraesCS1B02G208400	TraesCS2B02G269700	TraesCS3B02G260900	TraesCS2D02G465300	TraesCS2D02G465300	TraesCS1D02G315800	TraesCS1B02G037100
Chromosome	6A	1B	1B	2B	3B	2D	2D	1D	1B
Allele	C/G	A/G	C/T	A/G	G/T	A/G	C/A	C/T	A/G
Sequence	TGCAGACGGCGGCGGCGCGAACAAGCATGGTGGCGAAGCCGACACGGAGGAGGATCTCGGCGAG	TGCAGCAAGCTAAACCAAGTCTTGAAACTGGAGCAGCGAACAATTCTAAAGTCGCTTGAGGCTC	TGCAGCCAGAGCTCAAGAGGCCCCCTCGGCGGCATCTCAAAGGAGAAGGAAGGAGGAAGGCGAA	TGCAGCCGCCCGTCCACCCCCTTCGTCTTCGTCCAGCACCAACCCGTCGCCAGCGTCGCCGTCA	TGCAGCGTGTAGATTACCATGCTATTCACACAGAAGTTATCGGGTGTATATGTCATCAACTTTT	TGCAGGCAGCTGACTGTACTGGAGGGCCACACATTTATCGAAACCCTGCTGCCTGCCCTGGTTC	TGCAGGCAGCTGACTGTACTGGAGGGCCACACATTTATCGAAACCCTGCTGCCTGCCCTGGTTC	TGCAGGCATTCCATCAAGCAACCAGCAAGTCAGCACCAGAATCACCCCAATCAGACATGAGTGA	TGCAGGGGGTAGCCCTGAGCGCCGCCAAATCAGATCAAACGATGCCCTTCACTACATAAGCCAG
Marker	rs4500	rs12408	rs21674	rs25281	rs33960	rs46339	rs46340	rs46629	rs51316

*a: Triticum dicoccoides*, b: *Aegilops tauschii*, IT: Infection type, LP: Latent period.

## 4 DISCUSSION

The characteristics of the races used to evaluate the seedling stage and the effective and ineffective genes for each race are presented in Table 1. These races are known as the dominant black rust races in the country collected during 2015 and 2016 and with inoculated on 20 lines and differential cultivar of North American (Singh et al., 2008), the race of those has been determined. Isolate 95–2 was pathogenic to the *Sr31* resistance gene, which caused resistance to *Pgt* for a very long time. This isolate belonged to the Ug99 race group and was named TTKTK.

The results of the combined analysis of variance showed that in both traits of IT and LP, the highest total sum of squares was explained by the genotype effect (54.12% and 43.39%, respectively); therefore, it is inferred that genotypes have had a significant effect on data variation. After that, the highest justified variance of IT (36.33%) and LP (36.91%) was due to genotype–race interaction. The effect of the race for IT and LP explained 3.97% and 8.80% of the total variation, respectively. The small effect of the race indicates a relatively low diversity among the races under study.

The values of the genetic and phenotypic variation coefficients for ITs and LPs of all races were very close to each other. This indicates the high impact of genes on creating diversity among genotypes. Each of the phenotypic, genetic, and environmental diversity coefficients provides useful information about the observed genetic or environmental diversity. As seen in Table 3, the coefficient of phenotypic variation was higher than the genetic variation coefficient in all studied ITs and LPs. This is due to the influence of environmental factors. Since the phenotypic variance is derived from the sum of environmental and genetic variance, if the genetic variance is assumed constant, what causes one trait to differ in phenotypic and genetic variation would be environmental variance; that is, if the trait has high phenotypic variation and low genetic diversity, this indicates the effect of the environment. The higher the variation ratio of genetic to the environment, the greater the efficiency of selection, and therefore, favorable genotypes will be identified and selected more accurately from unfavorable ones.

The genetic variation coefficient reflects the variation among genotypes in terms of specificity understudy and cannot lonelily determine the extent of inheritance of this variation. This index, along with heritability, provides a good estimate of genetic progress in phenotypic selection (Burton & Devane, 1953). Simultaneous application of two very important parameters of heritability and genetic efficiency plays an important role in the development of varieties and genotypes. The high rate of genetic efficiency indicates additive gene action, and its low level represents nonadditive gene action. Genetic efficiency will not necessarily be high when estimated a high heritability for a trait. If high heritability and high genetic efficiency coincide, it will reflect the additive effects of genes; however, if high heritability is associated with low genetic function, it would indicate epistatic effects or dominance (Johnson et al., 1955). If trait heritability is < 0.2 (20%), it indicates low heritability, if it is between 0.2 (20%) to 0.5 (50%), it has moderate heritability, and if it is > 0.5 (50%) it has high heritability (Stansfield, 1991). High heritability with favorable genetic efficiency was observed in the PTRTF IT and LP, confirming the additive effects of the gene, and indicating that a large proportion of phenotypic variation explains the genotypic variation. High inheritance along with high genetic efficiency is a very important factor in predicting the effects of selecting the best individuals in a population.

Population structure has been used in genetic studies to explain the relationship among individuals "within populations" and "between populations" and shows a perspective on the evolutionary relationship of individuals in a population. Besides the existence of structure in a population that studied for association mapping, it is a deterrent to achieving reliable and positive results in a population that is not considered by the effects of population structure factors and relationships (Breseghello & Sorrells, 2006). Out of 282 genotypes in both groups of the original and imputed datasets, 215 genotypes (76.24%) belonged to three identified subpopulations and the remaining 67 genotypes (23.76%), including 22 variety and 45 landraces, were excluded from the mixed genotypes. The number of these genotypes in the mixed group was small indicating low mixing in the studied population. Allelic incorporation in landraces of more than one single gene cohort could have caused a slight mixing due to the wheat distribution in more than one ancestral population (Oliveira et al., 2012). The presence of gene flow from the introduction of new genotypes into farms previously can be another reason for mixing. In this regard, germplasm exchange between different Mediterranean regions due to the development of ancient empires is considered as another possible reason for mixing (Moragues et al., 2007). Geographical origin of genotypes, selection, and genetic drift cause subpopulation within a large population (Buckler IV & Thornsberry, 2002; Flint‐Garcia et al., 2003). The reason some varieties fall into associated groups of landrace is that some of the varieties have been selected and introduced based on indices from landrace and thus have a high genetic similarity to landrace (Alipour et al., 2017).

The extent of LD determines the number of markers required to identify marker–trait association as well as mapping resolution (Flint‐Garcia et al., 2003). On the other hand, in genome‐wide association mapping, locating QTLs is based on the extent of LD and is therefore of particular importance (Al‐Maskri et al., 2012). Generally, LD decreased with increasing genetic distance. The range of LD varied across chromosomes as well as from chromosomes to other chromosomes. The share of each of the A, B, and D genomes of the total original SNP markers was estimated to be in varieties and landraces, approximately 37, 46, and 17%, respectively, and in the imputed SNP markers, approximately 36, 50, and 13%, respectively. Nearly 87% of markers pairwise and 90% of significant markers pairwise in varieties and in landraces nearly 87% of markers pairwise and 88% of significant markers pairwise in original SNPs had genetic distance < 10 cM. In the imputed SNPs, all the pairwise markers and the significant pairwise markers related to the varieties and landraces were located < 10 cM. This issue indicants a high impact of LD on population, but LD in the population decreased (but not disappeared) with increasing distance between pairwise markers according to the results; this is due to other factors such as population structure, genetic drift, migration, selection, and mutation. As well as, the results showed higher LD values in varieties than landraces. This increase in LD in varieties can be attributed to the selection, whether natural or synthetic, which causes an LD among the selected and associated genes. Also, the selection of the ascending (or descending) trait controlled by two or more nonlinkage genes, despite the high genetic (physical) distance among the genes, increases the LD (Ataei et al., 2017). The relatively high level of LD in many chromosomal regions in the population indicates the usefulness and effectiveness of association mapping for identifying and confirming QTLs in these regions (Zhang et al., 2010).

Since association mapping was introduced in plants (Thornsberry et al., 2001), interest in the identification of new genes has gained popularity due to significant advances in DNA sequencing technology. The application of this method in plant populations to identify loci responsible for genetic variation including disease resistance is increasing (Hall et al., 2010; Kollers et al., 2013; Reich et al., 2001). Knowledge of the genetic diversity of wheat to accelerate genetic and breeding studies is essential to produce disease‐resistant genotypes because understanding the genetic nature of disease resistance can play a key role in the development of resistant genotypes. The use of genetic diversity of wheat germplasm to understand the genetic mechanisms of disease resistance through GWAS can be very effective in breeding programs aimed at producing resistant varieties of *Pgt*.

Association mapping introduced a total of 69 QTLs for the IT and 62 QTLs for the LP in the original dataset and the imputed dataset, 504 QTLs for the IT, and 454 QTLs for the LP with a 0.1% probability level (*p* ≤ .001). The highest number of QTLs identified for the IT of studied races in the original and imputed dataset was nonuniformly distributed on the B genome chromosomes.

After Bonferroni correction aimed to identify markers highly correlated with the target trait, the number of identified QTLs reduced in the original dataset, so the total of the QTLs for the studied races IT was 3 and for the LP 7 QTLs remained. In the imputed dataset, the number of QTLs of the IT was reduced to 10 QTLs and the number of QTLs of the LP decreased to 71 QTLs. To answer the question of whether QTLs identified with specific genomic regions in this study have been previously introduced or not, the results of this study were compared with previous findings. Some of the QTLs identified in this study corresponded to the genomic regions of the *Sr* genes and the QTLs reported in previous studies (Yu et al., 2011, 2012). B chromosomes were fined chromosomes carrying rust resistance genes in wheat, which has been reported in various studies (Aoun et al., 2016; Crossa et al., 2007; Letta et al., 2014; Yu et al., 2011). In the present study, only one marker (rs23510) was identified based on the results of the B genome located on chromosome 6 and was associated with the genomic locus that caused resistance to TTKTK. The number of markers in the B genome associated with the IT and the LP was significantly higher in the imputed dataset than in the original dataset and they were distributed on chromosomes 1B, 6B, 3B, 2B, and 5B, respectively. Letta *et al*. [19] identified a QTL on chromosome 1B for resistance to the JRCQC; this disease‐resistant genomic region has also been mentioned. The rs51316 marker associated with the TTTTF and PTRTF IT was largely close to the QTL identified by Letta et al. (2014). On the other hand, in this chromosomal position, the *Sr14* gene is resistant to some *Pgt* races in wheat (Singh et al., 2006), but a significant effect on the IT of each race was observed in this study. A specific study on TTKTK infection type belonging to the Ug99 group and reported in the study of Jin et al. (2007) did not show any significant effect at 106.99 cM located on chromosome 1B marker rs5923. This marker is related to the genomic region that caused seedling resistance to the TTTTF and was reported in the study by Letta et al. (2014). Not only the *Pgt* resistance gene is located in this region, but also the Lr46/Yr29/Pm39 gene block and the unknown resistance genes are in the adult plant stage (Bhavani et al., 2011; Singh et al., 2013). Several *Pgt* resistance genes, including *Sr9*, *Sr16*, and *Sr28* (McIntosh et al., 1995) and *SrWeb*, are located on the long arm of chromosome 2B. The *SrWeb* gene causes resistance to Ug99, while none of the four *Sr9* alleles has this ability (Jin et al., 2007). No resistance to TTKTK was found on chromosome 2B; the *SrWeb* gene may not be present in the studied germplasm, or there is no marker associated with this gene. After the B genome, the A genome had the highest marker–trait association. In the original dataset after Bonferroni correction, only one marker (rs22132) was identified in the A genome, located on chromosome 7 at 133.92 cM, and was associated with both PTRTF IT and LP characteristics. There were no QTLs in the A genome in the imputed SNPs for the IT of any of the studied races, but 8 QTL (1 QTL on 2A, 4 QTL on 3A, and 3 QTL on 6A) in TTTTF, 14 QTL (3 QTL on 1A, 1 QTL on 2A, 4 QTL on 3A and 6 QTL on 6A) in PTRTF, and 7 QTL (2 QTL on 3A, 1 QTL on 5A, 3 QTL on 6A and 1 QTL on 7a) in TTKTK were identified for the LP. TKTTF was not associated with genomic A chromosomes. The *Sr34* and *Sr38 Pgt* genes are located on chromosome 2A (Letta et al., 2014) and are somewhat close to the rs17477 marker in genomic position, but because the resistance of these two genes is broken by the studied races and as a result, they were inactivated against rust disease with no QTL associated region with the IT. Therefore, this marker can be used in marker‐assisted selection to identify *Sr34* and *Sr38*. The coexistence of two markers cfa2201 and wPt‐5839 with two *Sr34* and *Sr38 Pgt* resistance genes was reported in a study (Letta et al., 2014). These two genes are ineffective against the Ug99 race group, one of which (TTKTK) has been investigated in the present study (Jin et al., 2007; Singh et al., 2011). The *Sr7* and *SrND643* resistance genes (probably the *Sr7* allele) are located on the long arm of chromosome 4A (Basnet et al., 2015; McIntosh et al., 1995). This gene caused resistance to the PTRTF race, but no marker–trait association was found in this study. Other studies have also mapped genomic regions associated with resistance to black rust disease in the A genome such as a marker wpt‐734078 (Bhavani et al., 2011) and wpt‐6869 (Rouse et al., 2014) on chromosome 1AS.

The D genome had the lowest number of marker–trait associations. In the original dataset between the IT and the LP of all the studied races, only one QTL was identified for the TKTTF IT associated with the rs46629 marker on the chromosome 1D. In the imputed dataset, this marker retained its association with the TKTTF IT, and the only marker had the marker–trait association on all TKTTF D genome chromosomes. The TTKTK had no marker–trait association with the D genome. For the TTTTF IT, 2 QTLs were on the 2D and for the LP, 1, 3, and 2 QTLs were on 1D, 4D, and 7D, respectively. In PTRTF, 1, 3, and 3 QTLs were observed on 1D, 4D, and 7D, respectively. Edae et al. (2018) in their study reported QTHJC resistance‐related IWB17135 marker and TPMKC resistance‐related IWA2415 marker on the short arm of chromosome 2D. Tsilo et al. (2009) mapped a QTL on the chromosome 2D at 78.50 cM, which was 1.1 cm from the *Sr6 Pgt* resistance gene. In the original dataset, there were 6 QTLs on unknown chromosomes, 2 QTLs for TTTTF LP, 3 QTLs for PTRTF LP, and 1 QTL for TTKTK LP. In the imputed dataset, 2 QTLs associated with PTRTF LP and 1 QTL associated with TTKTK LP overlapped with the original dataset were identified.

The markers with the highest marker–trait association were blasted at the Ensembl database to identify overlapping genes, their molecular function, and biological processes, as well as to identify orthologous genes. The results indicated that the genes adjacent to the markers play an important role in biosynthetic pathways such as ion transport, redox, protein processing, phosphorylation, and so on. In general, environmental stresses cause significant changes in the expression levels of many of these genes in plants, so such changes result in the accumulation or reduction of important metabolites, changes in enzyme activity and protein synthesis rates, as well as the production of new proteins (Zhu, 2016) that deal with environmental stress in various ways. ROS, phospholipid‐derived signals, and cyclic nucleotide‐dependent signals, as well as some plant hormones, are involved in stress signaling (Ali et al., 2018; Jamei et al., 2018). In response to the pathogen attack, ROS is produced by the attacked plants, acts as an important sign of stress, and reduces the amount of stress damage possible by activating defense mechanisms (Ali et al., 2018; Jamei et al., 2018; Lehmann et al., 2015). Membrane lipids by regulating membrane fluidity and other physicochemical properties cause secondary signaling molecules in response to stress. Biosynthesis of lipids and their degrading enzymes plays several roles such as direct or indirect regulation or effect on stress signaling and tolerance (Jamei et al., 2018). In plants, a variety of biotic and abiotic stresses such as pathogens, drought, salinity, high temperature, intense light, hormones, and nodulation factors cause changes in cytosolic calcium levels and lead to stress transmission (Monihan, 2011; Pandey et al., 2015). In many transcriptional signaling pathways, the major form of signal transduction is reversible protein phosphorylation. Protein kinases such as mitogen‐activated protein kinase (MAPK) are essential in stress developmental, hormonal, biotic, and abiotic signaling. Protein phosphatases are responsible for dephosphorylating the phospho‐proteins. Protein phosphatases are subdivided into serine/threonine phosphatase, tyrosine phosphatase, and dual phosphatases through their specific substrates, which play an important role in serine/threonine phosphatase transcriptional stress transcription (Jamei et al., 2018). Eventually, protein kinases are likely to target transcription factors and bind to the stress‐responsive gene's promoter and consequently activate transcription (Jamei et al., 2018).

After identifying the nearest wheat gene or genes to the markers, orthologous genes with these genes were examined. Numerous orthologous genes were observed, but only genes with a high percentage of identity were selected. The identified orthologous genes were generally related to two species of *Triticum dicoccoides* and *Aegilops tauschii*, the ancestors of wheat, and they have introduced to it during the evolutionary stages of wheat by hybridization (Dvorak et al., 1998; Kerber, 1964; Kihara, 1944; Kislev, 1979; Matsuoka & Nasuda, 2004; McFadden & Sears, 1946). These orthologous genes had a high percentage of identity with the genes identified in wheat, which involved in stress resistance mechanisms.

## 5 CONCLUSION

Association mapping of resistance of Iranian bread wheat varieties and landraces to four *Pgt* races in the seedling stage in terms of LP and IT. The studied germplasm was grouped into three subpopulations. According to the results of the original and imputed dataset, the highest number of the pairwise marker in varieties and landraces was observed on the B genome. In the original dataset, the highest LD was observed in the D genome of varieties and landraces but in an imputed dataset, it was observed in the A genome of varieties and landraces. The results showed a significant difference in the LD value on different chromosomes. However, the varieties had higher LD compared with the landraces, which could be attributed to the selection made during the production and release stages of the varieties. Genome‐wide association study based on the original dataset revealed 69 and 62 marker–trait associations for races IT and their LP, respectively. The number of marker–trait associations in the imputed dataset was 504 for IT and 454 for the LP. When Bonferroni correction was performed, in the original dataset, two QTLs for the TTTTF LP, one QTL for the IT and four QTLs for the LP of PTRTF, one QTL for the IT, and one QTL for the LP of TTKTK, and one QTL for the TKTTF IT remained. In the imputed dataset, the TTTTF IT by six QTLs and its LP by 26 QTLs, the PTRTF IT, and the LP by 1 and 31 QTLs, respectively, the TTKTK LP by 14 QTLs, and the TKTTF IT by 3 QTLs was controlled. No marker–trait association was detected in the TTKTK IT and TKTTF LP. Nine markers out of 51 SNP markers (very significant) had a chromosomal position similar to the adjacent *Triticum aestivum* gene or genes. By studying the molecular function and biological processes of these genes, it was found that these genes have different mechanisms that cause stress resistance. The results obtained from this study can be important to facilitate and accelerate breeding programs through marker‐assisted selection.

## CONFLICT OF INTEREST

The authors declare that they do not have conflict of interest. The funding body was involved in the material creation, designing the study, data analysis, and writing the manuscript.

## Ethical Approval

Not applicable.
